# Exploring the impact of maternal early life adversity on interoceptive sensibility in pregnancy: implications for prenatal depression

**DOI:** 10.1007/s00737-024-01504-7

**Published:** 2024-08-19

**Authors:** Paul W. Savoca, Laura M. Glynn, Molly M. Fox, Misty C. Richards, Bridget L. Callaghan

**Affiliations:** 1 Department of Psychology, University of California, Psychology Building 1285, Box 951563, Los Angeles, CA 90095, USA; 2 Department of Psychology, Chapman University, Orange, USA; 3 Department of Anthropology, University of California, Los Angeles, USA; 4 Department of Psychiatry and Biobehavioral Sciences, University of California, Los Angeles, USA; 5 David Geffen School of Medicine, University of California, Los Angeles, CA, USA

**Keywords:** Pregnancy, Interoception, Depression, Early-life adversity

## Abstract

**Purpose:**

Pregnancy is a sensitive period of development in adult life characterized by massive changes in physical, emotional, and cognitive function. Such changes may be adaptive, e.g., facilitating adjustment to physical demands, but they may also reflect or contribute to risks inherent to this stage of life, e.g., prenatal depression. One cognitive ability that may undergo change during pregnancy and contribute to mental wellness is interoception - the ability to perceive, integrate, and model sensory information originating from the body. Strong interoceptive abilities are associated with lower rates of depression in non-pregnant adult populations, and interoception is generally weaker in individuals at higher risk for depression, for example, exposure to early life adversity (ELA). In the present online, cross-sectional study, we investigated whether interoception in pregnant women differed based on histories of ELA, in ways that increased their relative risk for prenatal depression symptoms.

**Methods:**

The pregnant individuals were in the second trimester of their first pregnancy and were compared to a group of nulliparous, non-parenting women.

**Results:**

Previous exposure to ELA significantly moderated pregnancy-related differences in self-reported interoception (interoceptive *sensibility*). A further moderated-mediation analysis revealed that the extent to which interoceptive sensibility buffered against depressive symptoms was conditional on ELA exposure, suggesting more ELA is associated with lower interoceptive sensibility during pregnancy, which increased prenatal depression risk.

**Conclusions:**

Together this work suggests that levels of interoception during pregnancy are sensitive to previous adversity exposure. It also suggests that interoceptive-focused interventions for preventing/treating prenatal depressive symptoms in high-risk women may be worth exploring.

## Introduction

Pregnancy is a period of both risk and resilience for mental wellness. While most women experience normative fluctuations in mood across pregnancy, a significant minority (~ 12%) will experience depression (Bennett et al. 2004; Newham and Martin 2013). Critically, depression risk during and shortly following pregnancy (i.e., perinatal) is not evenly distributed in the population, with women who have experienced early life adversity (ELA) being particularly vulnerable (Tebeka et al. 2021; Wajid et al. 2020). Left untreated, perinatal depression can have deleterious consequences for the mother, fetus/infant, and family (Meaney 2018; Sawyer et al. 2019), potentially perpetuating cycles of adversity across generations. Thus, discovery of new treatments and interventions for perinatal depression requires a detailed understanding of mechanistic pathways that might be impacted by a woman’s childhood experiences.

While several differences exist between pregnancy-related depression and major depressive disorder that occurs at other stages of the lifespan (Batt et al. 2020), both are characterized by core negative affective symptoms involving low or irritable mood, as well as abnormalities across numerous physiological systems (Aruldass et al. 2021; Dowlati et al. 2010; Juruena 2014; Lamers et al. 2013; Zeng et al. 2012). A compelling and empirically supported theory is that depression, and many depressive symptoms, are primarily characterized by an underlying issue of inefficient energy regulation (Allen et al. 2018; Barrett et al. 2016; Chan et al. 2019; Shaffer et al. 2022). Sustained dysregulated energy expenditure can be experienced as negative affect (Barrett 2017; Shaffer et al. 2022). Critically, efficient energy regulation is supported by interoception (Quigley et al. 2021; Sennesh et al. 2022; Shaffer et al. 2023), the process by which the brain perceives, integrates, and models sensory information generated from within the body (Craig 2003; Khalsa et al. 2018; Sherrington 1952). Such theories thus suggest altered interoception is a critical mechanism involved in depression (Barrett et al. 2016; Khalsa et al. 2018). As metabolic demands are drastically increased during pregnancy and postpartum, interoception may be an important pathway via which elevated peripartum depression risk is realized or mitigated. If so, this would suggest potential efficacy of interoception-targeted treatments for depressed women during the prenatal period.

To date, we are aware of four studies that assessed pregnancy-related changes in interoception, with most noting improvement in interoceptive sensibility – the self-perceived ability to focus on or be cognizant of internal bodily sensations (Critchley and Garfinkel 2017; Garfinkel et al. 2015; Suksasilp and Garfinkel 2022). In one study (*N* = 134) women were assessed across gestation and into the postpartum period (Singh Solorzano et al. 2022); interoceptive sensibility was generally greater during pregnancy, relative to postpartum, and higher interoceptive sensibility during early pregnancy was associated with lower postpartum depression. A second study (*N* = 500) found pregnant (any trimester) women to have greater interoceptive sensibility scores (domain of not ignoring physical sensations) than non-pregnant (though not necessarily childless) women (Crossland et al. 2022). A follow-up to this study also found that interoceptive sensibility during pregnancy was important for antenatal attachment (Stafford et al. 2024). A final study (*N* = 32) found that primiparous 2nd − 3rd trimester mothers had lower interoceptive sensibility scores on a sub-domain related to the ability to attend to bodily sensations compared to multiparous 2nd −3rd trimester mothers, but no association with depressive symptoms (Noda et al. 2022), suggesting interoceptive sensibility may increase with parity. While these studies paint a general picture of improvements in interoception during pregnancy, some are limited by the lack of a non-pregnant control group, and none assess interoception in groups who are at higher risk for peripartum depression – ELA-exposed mothers. As interoceptive predictions are learned and refined across development (Atzil and Barrett 2017), adverse early environments, often characterized by uncertainty (Davis and Glynn 2024; Ellis et al. 2022), have the potential to impair interoceptive predictions, increasing depression risk across the lifespan. Indeed, several studies have shown that self-reported interoception is lower in ELA exposed than non-exposed adults (not pregnant; Schaan et al. 2019) and that ELA exposed individuals are at heightened risk of depression both before (Aran et al. 2024) and during pregnancy (Tebeka et al. 2021). As such, ELA exposure may be a critical variable to consider when attempting to understand individual differences in interoception within pregnant women and may also explain variability in prenatal depressive symptoms.

To address these outstanding questions, we examined interoceptive sensibility among first-time pregnant women and nulliparous women of a similar age who were not parenting any children, with varying levels of ELA exposure. We first tested whether ELA exposure moderated pregnancy-related differences in interoceptive sensibility. For statistically significant interactions, we then used moderated-mediation to test if there was an indirect association between pregnancy and depression symptoms via interoceptive sensibility that was conditional on ELA exposure. Based on the prior literature noting general improvements in self-reported interoception in pregnancy (Singh Solorzano et al. 2022), we predicted that pregnancy would be associated with higher levels of interoceptive sensibility, and we hypothesized that this would be especially evident for women with low levels of ELA. We also hypothesized that there would be an indirect association between pregnancy and depression symptoms through interoceptive sensibility that would be conditional on ELA exposure. That is, for women with relatively lower exposure to ELA, we expected that pregnancy would be associated with better interoceptive sensibility, which would in turn be associated with fewer depressive symptoms. Alternatively, for women with relatively higher ELA exposure, we expected that pregnancy would be associated with worse interoceptive sensibility, which would in turn be associated with greater depressive symptoms.

## Materials and methods

### Study design

Data for this cross-sectional analysis were collected during the first wave of a longitudinal online study of interoception in pregnant women. Participants were 18-42-year-old females who had never parented any children and self-identified as being in the second trimester of their first pregnancy carried past 8 weeks at wave 1 (Pregnant group) or were women who had never been pregnant (past 8 weeks) and were not currently pregnant (Comparison group). Recruitment involved targeted advertising on social media sites (e.g., Instagram), as well as use of participant research panels (i.e., Prolific). All measures were collected online using the Gorilla.sc website. All research procedures were approved by the University of California, Los Angeles (UCLA) Institutional Review Board.

### Recruitment and screening

Given the online data collection method, we employed systematic and rigorous data quality checking procedures, including two attention checks, based on a priori defined exclusion criteria (for details see Supplemental Materials).

After an initial enrollment of *N* = 229 participants, our quality checking procedures removed 37 participants. Our final sample for analysis was *N* = 192 (*n* = 75 Pregnant, *n* = 117 Comparison). Groups differed in mean age, income, and education composition (Table 1), so we adjusted for age, income, and education in all analyses. Distribution of race did not statistically differ between the pregnant and not pregnant groups, but was trending, so we also adjusted for race in all analyses.

### Questionnaires

The *Edinburgh Postnatal Depression Scale (EPDS)* is a 10-item scale intended to measure depressive symptoms (Cox et al. 1987), validated for use in both pregnant and non-pregnant women (Bergink et al. 2011; Cox et al. 1996). We excluded one item of the EPDS that assessed suicidality, as per common practice in studies without clinical follow-up, and the 9- and 10-item versions are highly correlated (Qiu et al. 2023). We calculated the total score using the sum of scores for each response to the 9-items, resulting in a maximum total score (of 27). The EPDS also suggests a scoring cutoff for “probable depression” as a total score ≥ 13 (Matthey et al. 2006; Murray and Cox 1990). The measure showed good reliability in our sample (*α* = 0.86).

While interoception comprises several distinct dimensions, in the current study we focused on interoceptive *sensibility*, which is the self-perceived ability to focus on, or be cognizant of internal bodily sensations (Garfinkel et al. 2015). To test for overall differences in interoceptive sensibility we used the 21-item *Interoceptive Accuracy Scale (IAS)*, which probes participants self-perceived accuracy (i.e., sensibility) in detecting physiological signals (Murphy et al. 2020). The IAS generates a total score (ranging 21–105). Reliability in the sample was good (*α* = 0.86). We also used the 37-item *Multidimensional Assessment of Interoceptive Awareness (MAIA-2)*, which examines eight distinct dimensions of interoceptive sensibility (rather than awareness, as the name suggests) with reliability ranging from acceptable to excellent in our sample (*Noticing* (*α* = 0.68), *Not-distracting* (*α* = 0.86), *Not-worrying* (*α* = 0.77), *Attention Regulation* (*α* = 0.88), *Emotional Awareness* (*α* = 0.84), *Self-regulation* (*α* = 0.85), *Body Listening* (*α* = 0.88), *Trusting* (*α* = 0.92); Mehling et al. 2018), despite not having been validated in pregnant women (Stafford et al. 2024). There is no interpretable combined total score on this measure, and as such we considered regressions using MAIA-2 subscales as separate families of comparison.

The *Childhood Trauma Questionnaire (CTQ)* asks participants about their experience with 6 domains of potentially traumatic events during the first 18 years of life, including physical, emotional, and sexual abuse, and physical and emotional neglect (Pennebaker and Susman 1988). Participants rated the intensity of each trauma experienced on a scale of 1 (*Not at all traumatic*) to 7 (*Extremely traumatic*). Following previously published scoring procedures (Ju et al. 2020), a total score of ELA was computed for each participant by summing the intensities of all reported traumatic experiences, which captures both the number of traumas experienced and their perceived impact. Given the CTQ contained only 6-items, each assessing different domains of trauma which should be largely independent from one another, we did not calculate an internal reliability, as it would not be meaningful.

### Analysis

We tested if the interaction between pregnancy status and ELA exposure was associated with the IAS and each of the MAIA-2 subscales of interoceptive sensibility using separate multiple linear regressions. For all models, we included age (continuous), participant race (categorical), participant income (categorical), and participant education (categorical) as covariates (for details on categorical covariates see Supplemental Materials).

In conditions where ELA interacted with pregnancy to explain variance in interoception, we examined whether pregnancy links with depressive symptoms were mediated by interoception, conditional on ELA. To do so, we conducted a moderated-mediation analysis (model 7) using PROCESS for R version 4.3 (Hayes 2017). Pregnancy status was a categorical independent variable, MAIA-2 subscale score was the mediator, CTQ scores moderated the association between pregnancy and interoceptive sensibility, and EPDS score was the outcome variable. To test the significance of the moderated-mediation, we used a nonparametric bootstrapping procedure of 5000 iterations. The full results of each model can be found in Supplemental Materials. All analyses were conducted using R statistical software (R Core Team 2016). All analysis scripts can be found: https://github.com/bablab/wiki_SoM/tree/main/scripts. Missing items were mean imputed for 10 participants (max 3 items per participant), see Supplemental methods for missing data handling. As this was an exploratory analysis, we did not conduct a power analysis to justify sample size, but instead used prior studies in pregnant women to guide sample size targets.

## Results

### Pregnancy group differences in depressive symptoms and ELA exposure

There were no group differences in depressive symptoms, proportions of participants with clinically elevated depression, or ELA exposure (see Supplemental Tables 1–3).

### Pregnancy associations with interoceptive sensibility, moderated by ELA exposure

**Fig. 1:**
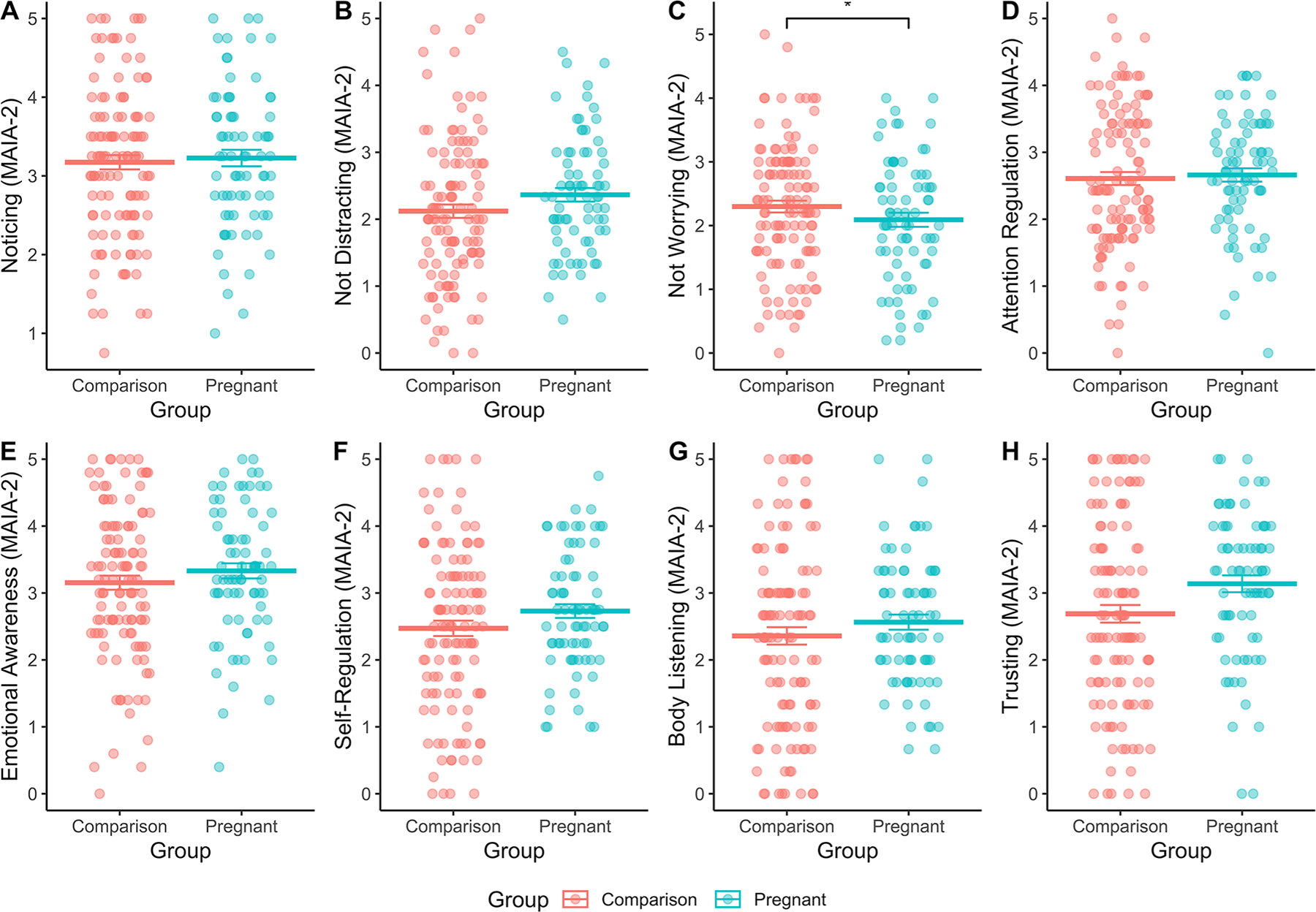
Pregnancy was associated with significantly lower levels of interoceptive Not Worrying, relative to the Comparison group, when controlling for ELA exposure (B = −0.19, SE = 0.08, *p* = 0.015). No other main effects of pregnancy on MAIA-2 subscales were significant, see Supplemental Tables 4–11 for statistics. This suggests that pregnant women worried more about bodily sensations compared to non-pregnant women

There were no group differences in interoceptive sensibility on the IAS, and no interactions between group and ELA on those scores (see Supplemental Table 2). For the MAIA-2 subscales, there was a significant main effect of group, but no interactions with ELA, on the MAIA-2 subscale – *Not-Worrying*, such that when controlling for ELA exposure, the pregnant group had a lower average score on the subscale than the comparison group (B = −0.19, SE = 0.08, *p* = 0.015), that is, the pregnant group were worrying more (Fig. 1, panel C).

**Fig. 2:**
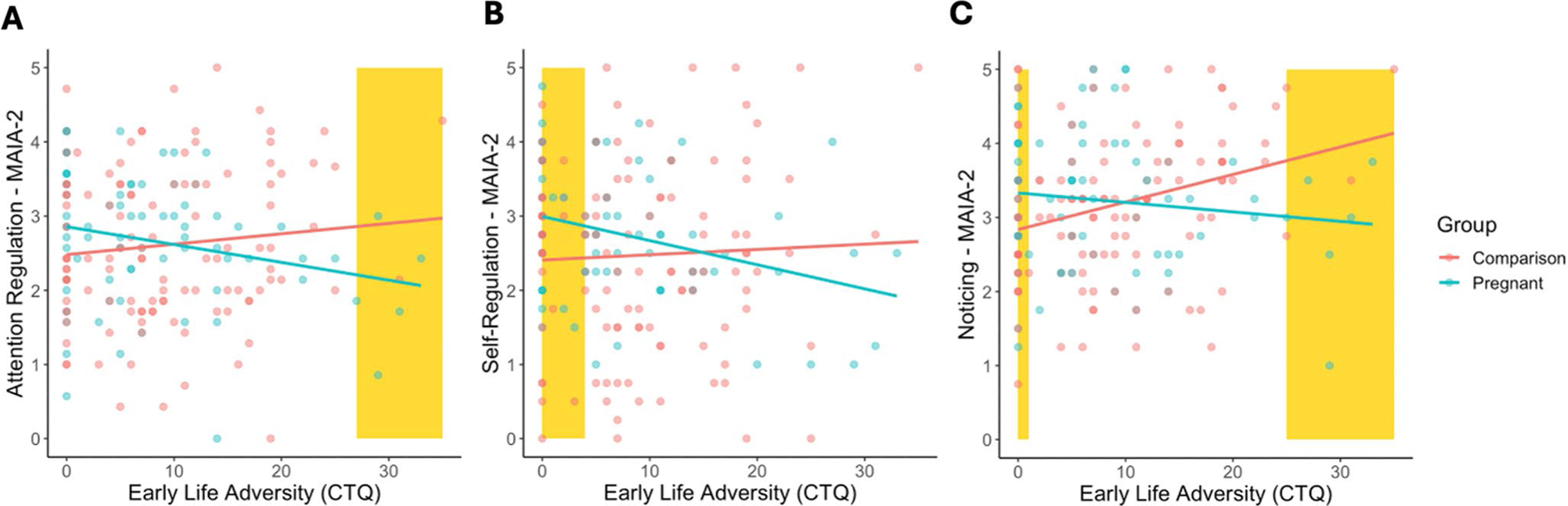
ELA exposure moderates the association between pregnancy and interoceptive sensibility. We found ELA-moderated differences between Pregnant and Comparison groups on the *Attention Regulation*, *Self-Regulation*, and *Noticing* subscales of MAIA-2 were significantly moderated by the experience of ELA. (**A**) *Attention Regulation* was negatively associated with ELA in pregnant women (B = −0.22, SE = 0.11, *p* = 0.045), but not comparison women (B = 0.13, SE = 0.08, *p* = 0.175). (**B**) *Self-Regulation* was negatively associated with ELA in pregnant women (B = −0.30, SE = 0.11, *p* = 0.005), but not comparison women (B = 0.11, SE = 0.12, *p* = 0.399). (**C**) *Noticing* was positively associated with ELA in comparison women (B = 0.26, SE = 0.09, *p* = 0.006), but not pregnant women (B = −0.15, SE = 0.11, *p* = 0.19). Regions of significant difference between groups are highlighted in yellow

There was a significant interaction between group and ELA on three MAIA-2 scales: *Attention Regulation* (B = −0.15, SE = 0.07, *p* = 0.038), *Self-Regulation* (B = −0.19, SE = 0.08, *p* = 0.023), and *Noticing* (B = −0.17, SE = 0.09, *p* = 0.014; Fig. 2; for full results of MAIA-2 interactions see Supplemental Tables 4–11). Though if conservative Bonferroni correction was applied these effects would not have survived correction. Post-hoc analyses indicated a significant negative association between *Attention Regulation* and ELA in the Pregnant group (B = −0.22, SE = 0.11, *p* = 0.045), but not Comparison group (B = 0.13, SE = 0.08, *p* = 0.175); a significant negative association between *Self-Regulation* and ELA in the Pregnant group (B = −0.30, SE = 0.11, *p* = 0.005), but not Comparison group (B = 0.11, SE = 0.12, *p* = 0.399); no association between *Noticing* and ELA in the Pregnant group (B = −0.15, SE = 0.11, *p* = 0.188), but a significant positive relationship in the Comparison group (B = 0.26, SE = 0.09, *p* = 0.006).

### Moderated-mediation of depressive symptoms

**Fig. 3:**
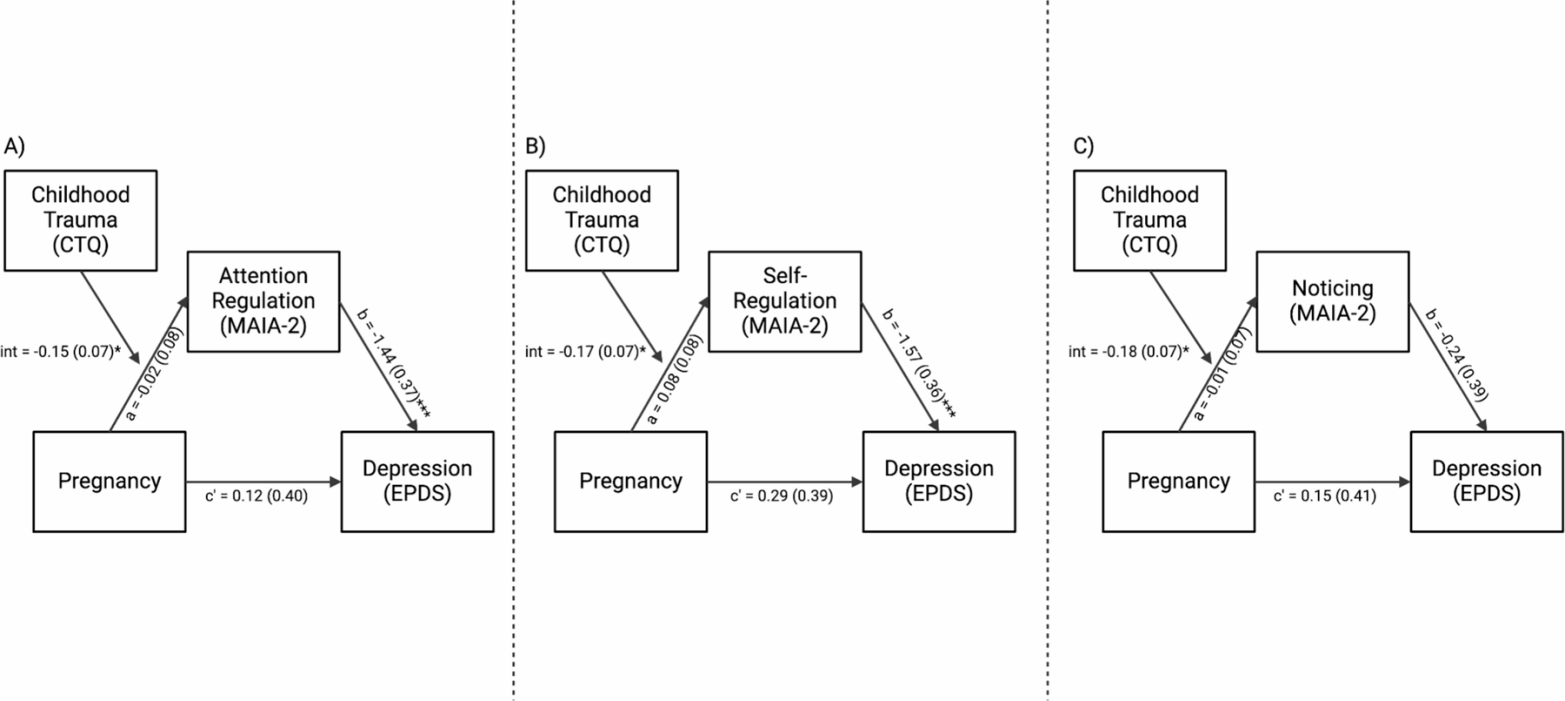
The mediation of pregnancy to depression symptoms was conditional on the level of exposure to ELA for *Attention Regulation* and *Self-regulation*, but not *Noticing.* (**A**) For *Attention Regulation*, the index of moderated mediation was significant (B = 0.22, SE = 0.12, 95%CI: [0.02, 0.49]). (**B**) For *Self-Regulation* the index of moderated mediation was also significant (B = 0.27, SE = 0.17, 95%CI: [0.02, 0.54]). (**C**) For *Noticing* the index of moderated mediation was not significant (B = 0.03, SE = 0.08, 95%CI: [−0.12, 0.23]). *p < 0.05; ***p < 0.001;

**Fig. 4:**
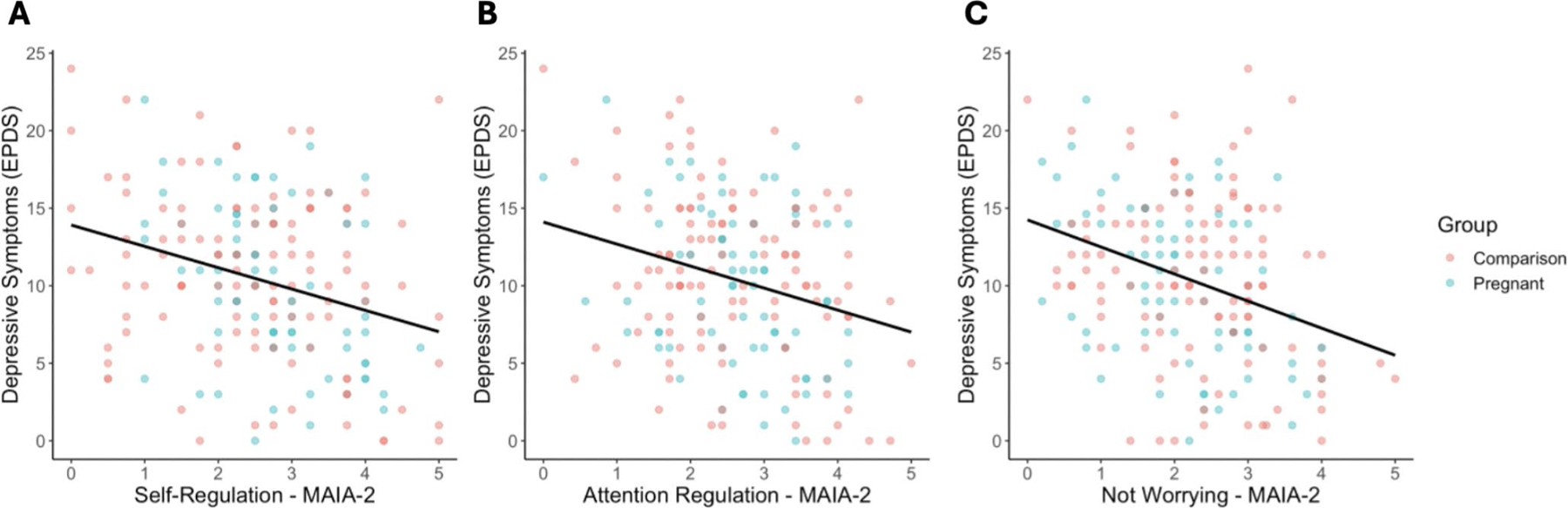
Greater interoceptive sensibility is associated with fewer depressive symptoms. (**A**) *Attention Regulation* was negatively associated with depressive symptoms, controlling for group (B = −1.44, SE = 0.37, *p* < 0.001). (**B**) Greater *Self-Regulation* was also associated with fewer depressive symptoms, controlling for group (B = −1.57, SE = 0.36, *p* < 0.001). (**C**) Greater Not Worrying was associated with significantly fewer depressive symptoms (B = −1.60, SE = 0.36, *p* < 0.001; Fig. 4C)

Given the exploratory nature of this study, we tested all MAIA-2 subscales in which an association with pregnancy was significantly moderated by ELA exposure (*Attention Regulation*, *Self-Regulation*, or *Noticing*), as mediators of a pregnancy to depression association in moderated-mediation models. In other words, these models tested whether the domain of interoception acted as a mediator between pregnancy and depression, conditional on exposure to ELA. For *Attention Regulation*, the index of moderated mediation was significant (B = 0.22, SE = 0.12, 95%CI: [0.02, 0.49]), suggesting that whether *Attention Regulation* acted as a mediator between pregnancy and depression symptoms was conditional on the level of exposure to ELA (Fig. 3A). A continuous analysis of conditional significance revealed that pregnant women with the lowest levels of ELA, did not have significantly different levels of *Attention Regulation* relative to non-pregnant women (B = 0.14, SE = 0.11, *p* = 0.194), while pregnant women with the highest levels of ELA, had significantly lower levels of *Attention Regulation* relative to non-pregnant women (B = −0.52, SE = 0.25, *p* = 0.042; Fig. 2A). Given *Attention Regulation* was negatively associated with depressive symptoms (B = −1.44, SE = 0.37, *p* < 0.001, Fig. 4A), this suggests that pregnant women exposed to high levels of ELA may be at greater risk for prenatal depressive symptoms via lower interoceptive sensibility (Supplemental Table 12). The index of moderated mediation for *Self-Regulation* was also significant (B = 0.27, SE = 0.17, 95%CI: [0.02, 0.54]; Fig. 3B), suggesting that whether *Self-Regulation* acted as a mediator between pregnancy and depression symptoms was conditional on the level of exposure to ELA. For *Self-Regulation* the continuous analysis of conditional significance demonstrated that pregnant women with the lowest levels of ELA had significantly greater levels of *Self-Regulation* relative to non-pregnant women (B = 0.27, SE = 0.11, *p* = 0.017), but pregnant and non-pregnant women did not significantly differ in *Self-Regulation* at the highest levels of ELA (B = −0.47, SE = 0.26, *p* = 0.069; Fig. 2B). Higher *Self-Regulation* was associated with lower levels of depressive symptoms (B = −1.57, SE = 0.36, *p* < 0.001, Fig. 4B), suggesting pregnant women with low levels of ELA may be protected from depressive symptoms via greater levels of interoceptive sensibility (Supplemental Table 13). For *Noticing*, the index of moderated mediation was not significant (B = 0.03, SE = 0.08, 95%CI: [−0.12, 0.23]; Fig. 3C), suggesting that pregnancy-related differences in interoceptive *Noticing* were not conditional on ELA exposure when controlling for depression symptoms, and did not mediate an association between pregnancy and depressive symptoms (Supplemental Table 14; see Fig. 2C for regions of significance).

Although ELA did not interact with pregnancy to moderate *Not Worrying* scores, pregnant women did score lower on the *Not Worrying* subscale (i.e., they worried more) when controlling for ELA. As such, we assessed whether *Not Worrying* was associated with depression. Indeed, lower *Not Worrying* scores (i.e., more worrying, which was characteristic of the pregnant group) was associated with higher depression (B = −1.60, SE = 0.36, *p* < 0.001; Fig. 4C).

## Discussion and conclusions

Several prior studies have noted improvements in interoceptive sensibility during pregnancy, relative to a non-pregnant control group or to the postpartum period (Crossland et al. 2022; Singh Solorzano et al. 2022). Here we tested whether any individual differences in interoception during pregnancy might be moderated by a mother’s experience with ELA, which is a known risk factor for peripartum depression (Tebeka et al. 2021), and appears to affect interoception in non-pregnant adults (Bonaz et al. 2021; Schaan et al. 2019). We observed that ELA significantly moderated the association between pregnancy and several domains of interoceptive sensibility. For domains encompassing the ability to sustain and control attention to body sensations (*Attention Regulation*) and the ability to regulate distress by attention to body sensations (*Self-Regulation*), greater levels of ELA were associated with lower interoceptive sensibility for pregnant women, but not the Comparison group. Specifically, for *Attention Regulation*, pregnant women did not differ from comparisons at low levels of ELA exposure but had significantly lower levels relative to comparisons after very high ELA exposure. In contrast, for *Self-Regulation*, pregnant women had significantly greater levels relative to comparisons in the context of low ELA exposure but did not significantly differ from comparisons after high ELA exposure. Greater interoceptive *Attention Regulation* and *Self-Regulation* were also associated with fewer depressive symptoms, suggesting a mechanism with the potential to buffer against depressive symptoms. As such, differences in interoception during pregnancy tied to ELA exposure may be an important risk mechanism by which adversity is transferred across generations.

An unexpected finding from this study was that pregnant women showed higher interoceptive worrying (lower scores on the *Not Worrying* subscale of the MAIA-2) than comparison women when controlling for ELA, but which was not moderated by exposure to ELA. It is possible that women may worry more about internal sensations during pregnancy because they are concerned about the health of the fetus and believe that these internal sensations might be signs of the health of the fetus. Interestingly, interoceptive worrying was also associated with greater depression across the entire sample, suggesting that worrying about interoceptive sensations might be a risk mechanism for increased peripartum depression risk for all women, regardless of ELA exposure. Additionally, we observed an unexpected positive association between childhood trauma and interoceptive *Noticing* in our Comparison group, which might be driven by higher attention to physical symptoms in the context of trauma. Exactly what factors drive this increase, and their implications for depression are open questions for future research, and highlight the need to better understand the complex relationship between ELA and later life interoception.

Given our findings that interoceptive sensibility in pregnancy is associated with depressive symptoms, it is important to consider the potential for intervening upon interoception as a novel treatment for peripartum depression. Indeed, prior studies have shown that interoception can be enhanced through a variety of non-invasive treatments (Nord and Garfinkel 2022; Weng et al. 2021), and behavioral interventions may be particularly appealing for pregnant women who wish to avoid fetal exposure to pharmacologic agents (Dennis and Chung-Lee 2006). For instance, mindfulness-based cognitive therapies have already been shown to reduce depressive symptoms in pregnant women (Matvienko-Sikar et al. 2016). Whether such mindfulness-based treatments would affect prenatal depressive symptoms via improvements in interoception, particularly for at-risk women, are critical topics for future investigations.

While this study provides novel insight into interoception during pregnancy, and relationships with early-life adversity, it is also important to consider some limitations. First, the prevalence of depressive symptoms is typically thought to be greater during pregnancy, but we did not find a significant difference in depressive symptoms between our groups. It is possible that we did not observe this elevated depression symptom incidence in pregnant women because of our sampling methods, which did not target depressed pregnant women and biased our sample to be highly motivated as they were both currently pregnant and still interested in completing research. Second, contrary to our hypothesis and previous work (Crossland et al. 2022; Singh Solorzano et al. 2022), we did not find substantial evidence for overall pregnancy-related improvements in interoceptive sensibility, that were not moderated by ELA exposure. We believe accounting for ELA exposure may explain this lack of replication, because we found interoception to be greater in the Pregnant group than Comparison group for women with low levels of ELA, but not high levels of ELA. Also, like many studies on maternal mental health, our sample was predominately white and well-educated. Future studies on this topic should be conducted in diverse groups of mothers, who may have different rates of experience with early adversity, interoception, and perinatal depression. Finally, the cross-sectional nature of this work, meant within-participant changes in interoception (i.e., before pregnancy to during pregnancy) could not be assessed. Additionally, participants reported their trimester of pregnancy and not specific week, which may have occluded within-trimester differences. Future longitudinal studies could be better positioned to elucidate whether pregnancy is associated with a boost in interoception.

Together the results from this study build on existing evidence that interoception may be an additional domain of phenotypic plasticity associated with the transition to motherhood with implications for psychopathology (Glynn et al. 2018). Furthermore, we provide new evidence that pregnancy-related individual differences in interoception can mediate the risk for prenatal depressive symptoms, conditional on the experience of ELA. We suggest continuing this direction of research can provide critical insight into novel and more effective treatments for depressive symptoms during pregnancy, by targeting interoceptive processing, particularly for mothers exposed to ELA.

## Supplementary Material

Supplementary Material

## Figures and Tables

**Table 1:** Sample characteristics, *N* = 192

	Pregnant (*n* = 75)	Comparison (*n* = 117)	Group Differences
Mean Age (SD)	27.97 (4.06)	25.73 (6.26)	*t*(190) = −3.02 *p* = 0.003
**Race (*n*)**			
American Indian/Alaska Native	0	3	X^2^(5, *n* = 192) = 9.80, *p =* 0.081
Asian	4	20	
Black or African American	8	14	
Multiple Races	5	11	
White	57	68	
Prefer Not to Answer	1	1	
**Household Income (*n*)**			
$0-50k	11	50	X^2^(6, *n* = 192) = 31.52 *p* < 0.001
$51-100k	31	41	
$101-150k	20	12	
$151-200k	7	6	
$201-250k	4	1	
$250k+	2	0	
Prefer Not to Answer	0	7	
**Education (*n*)**			
High School or equivalent	9	14	X^2^(6, *n* = 192 = 18.12, *p* = 0.006
Vocational/technical School	0	2	
Some College	11	43	
Bachelor’s Degree	35	42	
Master’s Degree	19	14	
Professional Degree	1	0	
Doctoral Degree	0	2	
***Characteristics (Mean (SD))***			
Depression (EPDS)	10.66 (5.38)	9.94 (5.05)	t(165) = 0.94 *p* = 0.349
EPDS “Probable Depression” (*n*)	25	43	X^2^(1, *n* = 192) = 0.11, *p* = 0.742
Childhood Trauma (CTQ)	9.07 (7.76)	8.15 (8.50)	t(148) = 0.76 *p* = 0.449
