# Supplementary material for: Exploring the impact of maternal early life adversity on interoceptive sensibility in pregnancy: implications for prenatal depression
Source: Arch Womens Ment Health. Author manuscript; Available in PMC 2025 Feb 6. (PMC11761834; doi:10.1007/s00737-024-01504-7)

**Supplemental Material**

***Systematic Data Quality Checking***

These criteria included removing any participants who: reported demographic information that did not meet the inclusion criteria (i.e., already having children, male sex, etc.; *n* = 29), completed the study in <5 minutes (*n* = 0), had multiple enrollments with the same email address (*n* = 0), failed at least one of two attention checks (*n* = 8), had incoherent or non-sensical answers (i.e., free responses that do not address the question; *n* = 0). The 5-minute exclusion completion time criteria was based on our own internal piloting of the study. We felt that any completion time below 5-minutes would not be humanly possible, and would likely reflect the use of an online bot. We also checked for outliers (+/- 3 SDs) for our primary measures of interest (IAS; MAIA-2; EPDS; *n* = 0).

***Missing Data Handling***

For any questions to which participants responded “prefer not to answer” on IAS, EPDS, or MAIA-2 we mean imputed the item value, using the individuals mean score on the scale (or subscale where applicable), after accounting for reverse scoring (no participant had more than 3 “prefer not to answer” responses across all questionnaires). For CTQ we did not mean impute “prefer not to answer” responses as to not assume traumatic experiences.

***Coding of Categorical Covariates***

We dummy coded the categorical covariates (race, income, and education) and we collapsed categories with very small sample sizes. For race, “American Indian” and “Prefer Not to Answer” were combined into “Other.” For income, all incomes $151,000 and greater were combined as a “$150k+” category, and the “Prefer Not to Answer” responses were included in $51-100k, as to fit the median income of our sample. For education, “Vocational School”, “Some College”, and “Bachelor’s Degree” were combined into “Higher Education”, while “Master’s Degree”, “Professional Degree”, and “Doctoral Degree” were combined into “Advanced Degrees.” All other categories were keep as listed in Table 1.

**Supplemental Table 1.** Full Model of Depressive Symptoms (EPDS) by Group

| **Predictor** | **B** | **SE** | ***t*** | ***p*** |
| --- | --- | --- | --- | --- |
| **(Intercept)** | 10.375 | 0.367 | 28.298 | 0.000 |
| **Group_num_c** | 0.144 | 0.411 | 0.351 | 0.726 |
| **participant_age_c** | -0.507 | 0.388 | -1.307 | 0.193 |
| **Income_cov_151+** | 0.136 | 0.449 | 0.303 | 0.762 |
| **Education_cov_HigherEd** | 0.172 | 0.457 | 0.375 | 0.708 |
| **Education_cov_HS** | 0.782 | 0.460 | 1.698 | 0.091 |
| **Race_cov_White** | -0.812 | 1.120 | -0.726 | 0.469 |
| **Race_cov_Asian** | -0.743 | 0.844 | -0.880 | 0.380 |
| **Race_cov_Black** | -1.115 | 0.810 | -1.376 | 0.170 |
| **Income_cov_0-50k** | 1.522 | 0.556 | 2.740 | 0.007 |
| **Income_cov_51-100k** | 0.773 | 0.543 | 1.422 | 0.157 |
| **Race_cov_Multiple** | -0.923 | 0.729 | -1.267 | 0.207 |
| R^2^/Adjusted R^2^ | 0.118/0.064 | | | |

**Note:** Using the cut-off scores for “probable depression” there was also no group difference (X^2^(1, n=192) = 0.108, p = 0.742)

**Supplemental Table 2.** Full Model of Interoceptive Sensibility (IAS) by Group

| **Predictor** | **B** | **SE** | ***t*** | ***p*** |
| --- | --- | --- | --- | --- |
| **(Intercept)** | 83.041 | 0.743 | 111.694 | 0.000 |
| **Group_num_c** | -0.206 | 0.834 | -0.247 | 0.805 |
| **participant_age_c** | -0.063 | 0.786 | -0.080 | 0.936 |
| **Income_cov_151+** | -0.624 | 0.910 | -0.685 | 0.494 |
| **Education_cov_HigherEd** | -0.745 | 0.927 | -0.804 | 0.423 |
| **Education_cov_HS** | -0.469 | 0.934 | -0.502 | 0.616 |
| **Race_cov_White** | -0.141 | 2.270 | -0.062 | 0.950 |
| **Race_cov_Asian** | -0.107 | 1.712 | -0.062 | 0.950 |
| **Race_cov_Black** | 1.069 | 1.644 | 0.651 | 0.516 |
| **Income_cov_0-50k** | -1.119 | 1.126 | -0.993 | 0.322 |
| **Income_cov_51-100k** | -1.662 | 1.101 | -1.509 | 0.133 |
| **Race_cov_Multiple** | -1.175 | 1.477 | -0.795 | 0.428 |
| R^2^/Adjusted R^2^ | 0.038/-0.020 | | | |

**Supplemental Table 3.** Full Model of Childhood Trauma by Group

| **Predictor** | **B** | **SE** | ***t*** | ***p*** |
| --- | --- | --- | --- | --- |
| **(Intercept)** | 8.708 | 0.580 | 15.020 | 0.000 |
| **Group_num_c** | 0.057 | 0.650 | 0.088 | 0.930 |
| **participant_age_c** | -0.618 | 0.613 | -1.008 | 0.315 |
| **Income_cov_151+** | -0.279 | 0.710 | -0.392 | 0.695 |
| **Education_cov_HigherEd** | 0.960 | 0.723 | 1.328 | 0.186 |
| **Education_cov_HS** | 1.129 | 0.728 | 1.551 | 0.123 |
| **Race_cov_White** | -1.232 | 1.771 | -0.696 | 0.487 |
| **Race_cov_Asian** | -1.367 | 1.335 | -1.024 | 0.307 |
| **Race_cov_Black** | -1.364 | 1.282 | -1.064 | 0.289 |
| **Income_cov_0-50k** | 0.932 | 0.878 | 1.061 | 0.290 |
| **Income_cov_51-100k** | 0.715 | 0.859 | 0.832 | 0.407 |
| **Race_cov_Multiple** | -0.586 | 1.152 | -0.508 | 0.612 |
| R^2^/Adjusted R^2^ | 0.060/0.002 | | | |

**Supplemental Table 4.** Full Model *Attention Regulation* by Group and Trauma Interaction

| **Predictor** | **B** | **SE** | ***t*** | ***p*** |
| --- | --- | --- | --- | --- |
| **(Intercept)** | 2.620 | 0.068 | 38.333 | 0.000 |
| **Group_num_c** | -0.023 | 0.077 | -0.297 | 0.767 |
| **Cumulative_Trauma_c** | -0.005 | 0.071 | -0.074 | 0.941 |
| **participant_age_c** | 0.003 | 0.072 | 0.042 | 0.967 |
| **Income_cov_151+** | -0.147 | 0.084 | -1.760 | 0.080 |
| **Education_cov_HigherEd** | 0.026 | 0.086 | 0.304 | 0.761 |
| **Education_cov_HS** | -0.100 | 0.087 | -1.150 | 0.252 |
| **Race_cov_White** | -0.370 | 0.209 | -1.774 | 0.078 |
| **Race_cov_Asian** | -0.415 | 0.158 | -2.631 | 0.009 |
| **Race_cov_Black** | -0.207 | 0.152 | -1.360 | 0.176 |
| **Income_cov_0-50k** | -0.163 | 0.104 | -1.574 | 0.117 |
| **Income_cov_51-100k** | -0.144 | 0.102 | -1.410 | 0.160 |
| **Race_cov_Multiple** | -0.386 | 0.136 | -2.835 | 0.005 |
| **Interaction** | -0.148 | 0.071 | -2.092 | 0.038 |
| R^2^/Adjusted R^2^ | 0.126/0.062 | | | |

**Supplemental Table 5.** Full Model *Noticing* by Group and Trauma Interaction

| **Predictor** | **B** | **SE** | ***t*** | ***p*** |
| --- | --- | --- | --- | --- |
| **(Intercept)** | 3.185 | 0.066 | 48.482 | 0.000 |
| **Group_num_c** | 0.026 | 0.074 | 0.355 | 0.723 |
| **Cumulative_Trauma_c** | 0.113 | 0.068 | 1.662 | 0.098 |
| **participant_age_c** | -0.030 | 0.070 | -0.437 | 0.663 |
| **Income_cov_151+** | -0.126 | 0.080 | -1.567 | 0.119 |
| **Education_cov_HigherEd** | 0.150 | 0.082 | 1.830 | 0.069 |
| **Education_cov_HS** | 0.052 | 0.083 | 0.624 | 0.534 |
| **Race_cov_White** | -0.263 | 0.201 | -1.312 | 0.191 |
| **Race_cov_Asian** | -0.328 | 0.152 | -2.167 | 0.032 |
| **Race_cov_Black** | -0.141 | 0.146 | -0.963 | 0.337 |
| **Income_cov_0-50k** | -0.105 | 0.100 | -1.054 | 0.294 |
| **Income_cov_51-100k** | -0.190 | 0.098 | -1.931 | 0.055 |
| **Race_cov_Multiple** | -0.200 | 0.131 | -1.532 | 0.127 |
| **Interaction** | -0.170 | 0.068 | -2.495 | 0.014 |
| R^2^/Adjusted R^2^ | 0.145/0.082 | | | |

**Supplemental Table 6.** Full Model *Trusting* by Group and Trauma Interaction

| **Predictor** | **B** | **SE** | ***t*** | ***p*** |
| --- | --- | --- | --- | --- |
| **(Intercept)** | 2.861 | 0.093 | 30.658 | 0.000 |
| **Group_num_c** | 0.131 | 0.105 | 1.250 | 0.213 |
| **Cumulative_Trauma_c** | -0.209 | 0.097 | -2.165 | 0.032 |
| **participant_age_c** | 0.006 | 0.099 | 0.063 | 0.950 |
| **Income_cov_151+** | -0.030 | 0.114 | -0.266 | 0.791 |
| **Education_cov_HigherEd** | 0.000 | 0.117 | 0.004 | 0.997 |
| **Education_cov_HS** | 0.035 | 0.118 | 0.296 | 0.768 |
| **Race_cov_White** | -0.567 | 0.285 | -1.991 | 0.048 |
| **Race_cov_Asian** | -0.518 | 0.215 | -2.405 | 0.017 |
| **Race_cov_Black** | -0.322 | 0.208 | -1.549 | 0.123 |
| **Income_cov_0-50k** | -0.249 | 0.142 | -1.760 | 0.080 |
| **Income_cov_51-100k** | -0.028 | 0.140 | -0.200 | 0.842 |
| **Race_cov_Multiple** | -0.290 | 0.186 | -1.561 | 0.120 |
| **Interaction** | -0.058 | 0.097 | -0.599 | 0.550 |
| R^2^/Adjusted R^2^ | 0.108/0.042 | | | |

**Supplemental Table 7.** Full Model *Emotional Awareness* by Group and Trauma Interaction

| **Predictor** | **B** | **SE** | ***t*** | ***p*** |
| --- | --- | --- | --- | --- |
| **(Intercept)** | 3.217 | 0.075 | 42.793 | 0.000 |
| **Group_num_c** | 0.102 | 0.084 | 1.216 | 0.225 |
| **Cumulative_Trauma_c** | 0.090 | 0.078 | 1.159 | 0.248 |
| **participant_age_c** | -0.136 | 0.080 | -1.710 | 0.089 |
| **Income_cov_151+** | -0.222 | 0.092 | -2.419 | 0.017 |
| **Education_cov_HigherEd** | 0.144 | 0.094 | 1.533 | 0.127 |
| **Education_cov_HS** | 0.065 | 0.095 | 0.679 | 0.498 |
| **Race_cov_White** | -0.269 | 0.230 | -1.174 | 0.242 |
| **Race_cov_Asian** | -0.358 | 0.173 | -2.066 | 0.040 |
| **Race_cov_Black** | -0.139 | 0.167 | -0.830 | 0.408 |
| **Income_cov_0-50k** | -0.152 | 0.114 | -1.332 | 0.185 |
| **Income_cov_51-100k** | -0.041 | 0.112 | -0.368 | 0.713 |
| **Race_cov_Multiple** | -0.273 | 0.150 | -1.822 | 0.070 |
| **Interaction** | -0.103 | 0.078 | -1.319 | 0.189 |
| R^2^/Adjusted R^2^ | 0.133/0.070 | | | |

**Supplemental Table 8.** Full Model *Body Listening* by Group and Trauma Interaction

| **Predictor** | **B** | **SE** | ***t*** | ***p*** |
| --- | --- | --- | --- | --- |
| **(Intercept)** | 2.429 | 0.090 | 26.904 | 0.000 |
| **Group_num_c** | 0.055 | 0.101 | 0.542 | 0.588 |
| **Cumulative_Trauma_c** | -0.058 | 0.093 | -0.625 | 0.533 |
| **participant_age_c** | -0.011 | 0.096 | -0.113 | 0.911 |
| **Income_cov_151+** | -0.111 | 0.110 | -1.003 | 0.317 |
| **Education_cov_HigherEd** | 0.103 | 0.113 | 0.914 | 0.362 |
| **Education_cov_HS** | 0.107 | 0.114 | 0.935 | 0.351 |
| **Race_cov_White** | -0.306 | 0.276 | -1.109 | 0.269 |
| **Race_cov_Asian** | -0.412 | 0.208 | -1.980 | 0.049 |
| **Race_cov_Black** | -0.249 | 0.201 | -1.239 | 0.217 |
| **Income_cov_0-50k** | -0.139 | 0.137 | -1.012 | 0.313 |
| **Income_cov_51-100k** | 0.087 | 0.135 | 0.647 | 0.519 |
| **Race_cov_Multiple** | -0.260 | 0.180 | -1.447 | 0.150 |
| **Interaction** | -0.183 | 0.094 | -1.949 | 0.053 |
| R^2^/Adjusted R^2^ | 0.082/0.015 | | | |

**Supplemental Table 9.** Full Model *Self-Regulation* by Group and Trauma Interaction

| **Predictor** | **B** | **SE** | ***t*** | ***p*** |
| --- | --- | --- | --- | --- |
| **(Intercept)** | 2.562 | 0.080 | 32.150 | 0.000 |
| **Group_num_c** | 0.093 | 0.089 | 1.038 | 0.301 |
| **Cumulative_Trauma_c** | -0.068 | 0.082 | -0.819 | 0.414 |
| **participant_age_c** | -0.027 | 0.085 | -0.325 | 0.746 |
| **Income_cov_151+** | -0.074 | 0.097 | -0.759 | 0.449 |
| **Education_cov_HigherEd** | 0.043 | 0.100 | 0.430 | 0.667 |
| **Education_cov_HS** | -0.014 | 0.101 | -0.142 | 0.887 |
| **Race_cov_White** | -0.208 | 0.243 | -0.853 | 0.395 |
| **Race_cov_Asian** | -0.241 | 0.184 | -1.308 | 0.193 |
| **Race_cov_Black** | -0.073 | 0.177 | -0.410 | 0.682 |
| **Income_cov_0-50k** | -0.098 | 0.121 | -0.811 | 0.418 |
| **Income_cov_51-100k** | 0.073 | 0.119 | 0.610 | 0.543 |
| **Race_cov_Multiple** | -0.316 | 0.159 | -1.993 | 0.048 |
| **Interaction** | -0.190 | 0.083 | -2.297 | 0.023 |
| R^2^/Adjusted R^2^ | 0.103/0.038 | | | |

**Supplemental Table 10.**  Full Model *Not-Worrying* by Group and Trauma Interaction

| **Predictor** | **B** | **SE** | ***t*** | ***p*** |
| --- | --- | --- | --- | --- |
| **(Intercept)** | 2.211 | 0.070 | 31.494 | 0.000 |
| **Group_num_c** | -0.193 | 0.079 | -2.453 | 0.015 |
| **Cumulative_Trauma_c** | -0.106 | 0.073 | -1.457 | 0.147 |
| **participant_age_c** | 0.152 | 0.074 | 2.045 | 0.042 |
| **Income_cov_151+** | -0.061 | 0.086 | -0.709 | 0.479 |
| **Education_cov_HigherEd** | -0.134 | 0.088 | -1.531 | 0.128 |
| **Education_cov_HS** | -0.025 | 0.089 | -0.279 | 0.780 |
| **Race_cov_White** | -0.071 | 0.214 | -0.332 | 0.740 |
| **Race_cov_Asian** | -0.022 | 0.162 | -0.139 | 0.890 |
| **Race_cov_Black** | -0.046 | 0.156 | -0.295 | 0.769 |
| **Income_cov_0-50k** | -0.177 | 0.107 | -1.663 | 0.098 |
| **Income_cov_51-100k** | -0.127 | 0.105 | -1.210 | 0.228 |
| **Race_cov_Multiple** | -0.045 | 0.140 | -0.320 | 0.749 |
| **Interaction** | -0.108 | 0.073 | -1.484 | 0.139 |
| R^2^/Adjusted R^2^ | 0.099/0.033 | | | |

**Supplemental Table 11.** Full Model *Not-Distracting* by Group and Trauma Interaction

| **Predictor** | **B** | **SE** | ***t*** | ***p*** |
| --- | --- | --- | --- | --- |
| **(Intercept)** | 2.215 | 0.071 | 31.216 | 0.000 |
| **Group_num_c** | 0.101 | 0.079 | 1.277 | 0.203 |
| **Cumulative_Trauma_c** | -0.094 | 0.074 | -1.275 | 0.204 |
| **participant_age_c** | 0.193 | 0.075 | 2.572 | 0.011 |
| **Income_cov_151+** | 0.065 | 0.087 | 0.749 | 0.455 |
| **Education_cov_HigherEd** | 0.113 | 0.089 | 1.281 | 0.202 |
| **Education_cov_HS** | -0.009 | 0.090 | -0.102 | 0.919 |
| **Race_cov_White** | -0.256 | 0.216 | -1.184 | 0.238 |
| **Race_cov_Asian** | -0.020 | 0.163 | -0.121 | 0.904 |
| **Race_cov_Black** | -0.151 | 0.157 | -0.960 | 0.339 |
| **Income_cov_0-50k** | -0.055 | 0.108 | -0.514 | 0.608 |
| **Income_cov_51-100k** | 0.024 | 0.106 | 0.225 | 0.823 |
| **Race_cov_Multiple** | -0.090 | 0.141 | -0.638 | 0.524 |
| **Interaction** | 0.013 | 0.074 | 0.174 | 0.862 |
| R^2^/Adjusted R^2^ | 0.120/0.055 | | | |

**Supplemental Table 12.** Attn. Reg Mod-Med


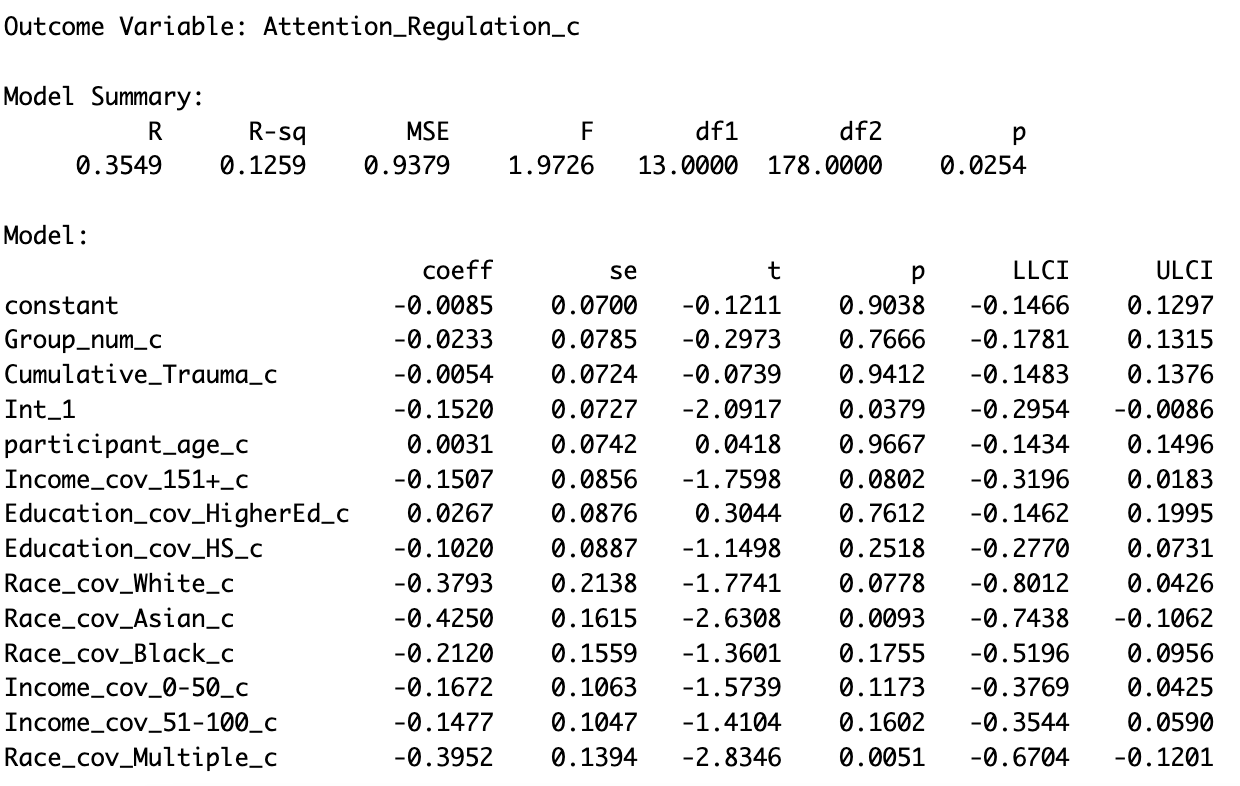


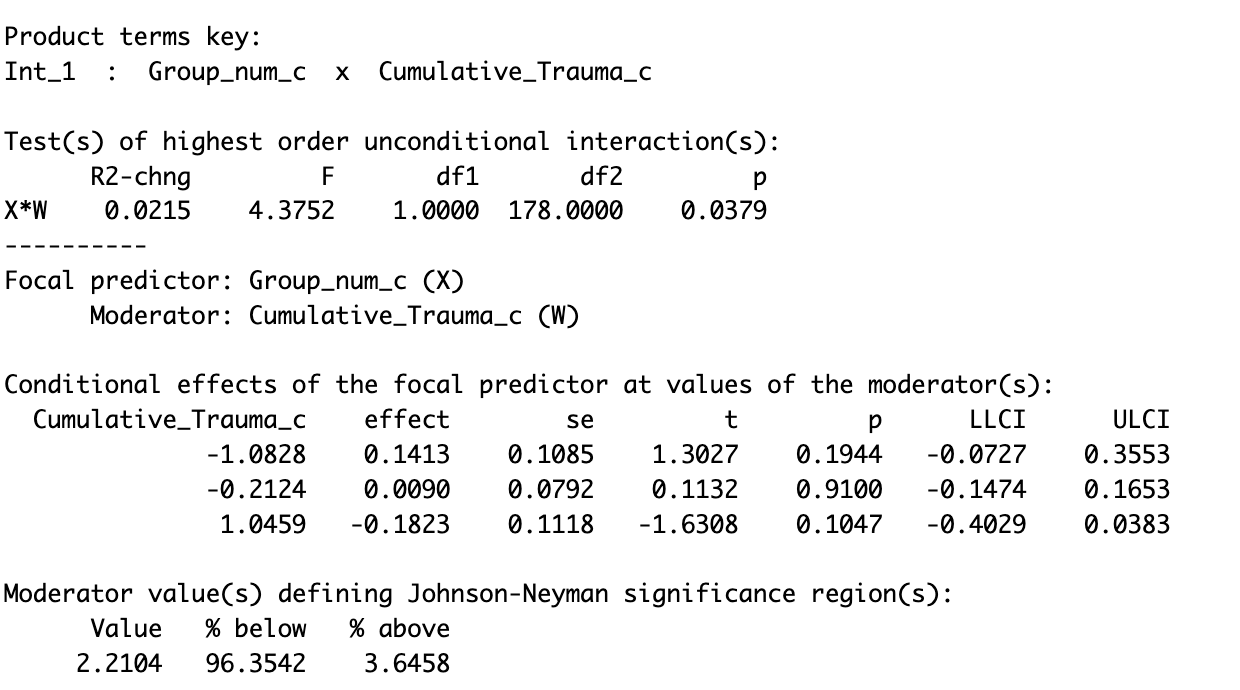


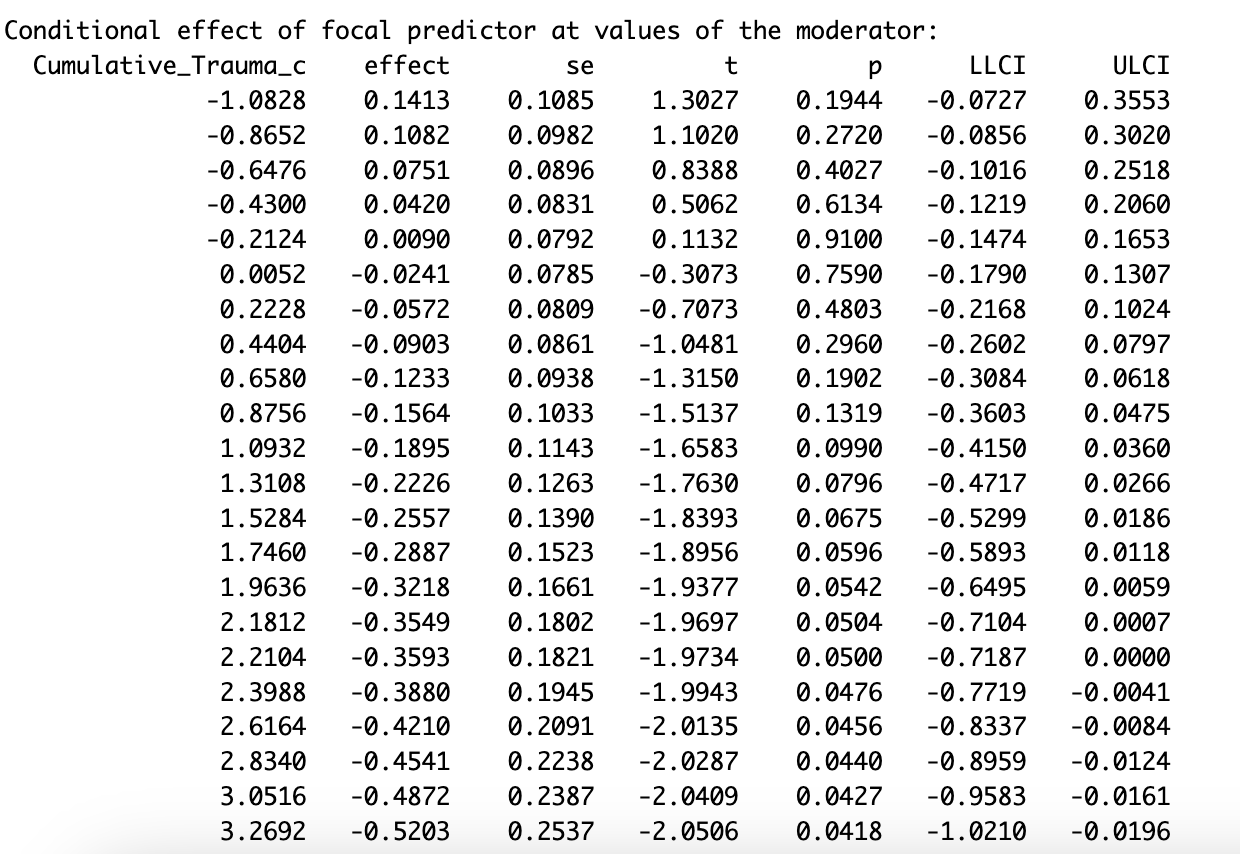


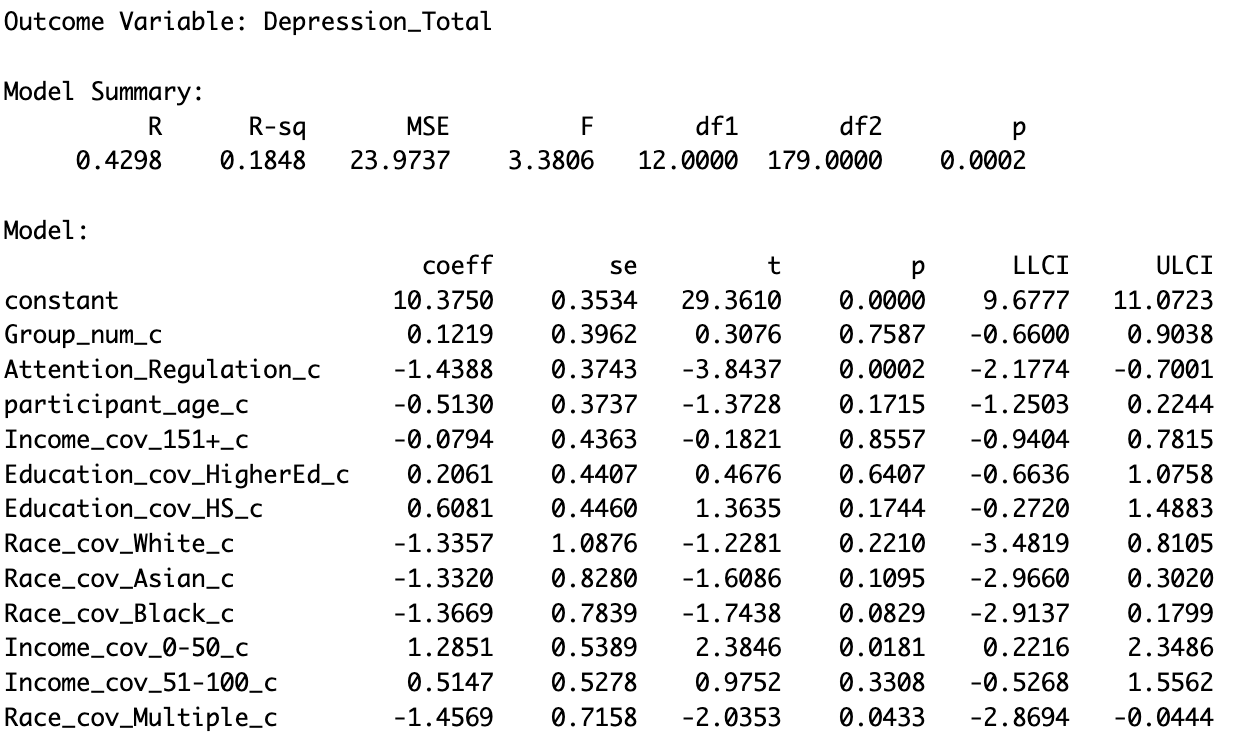


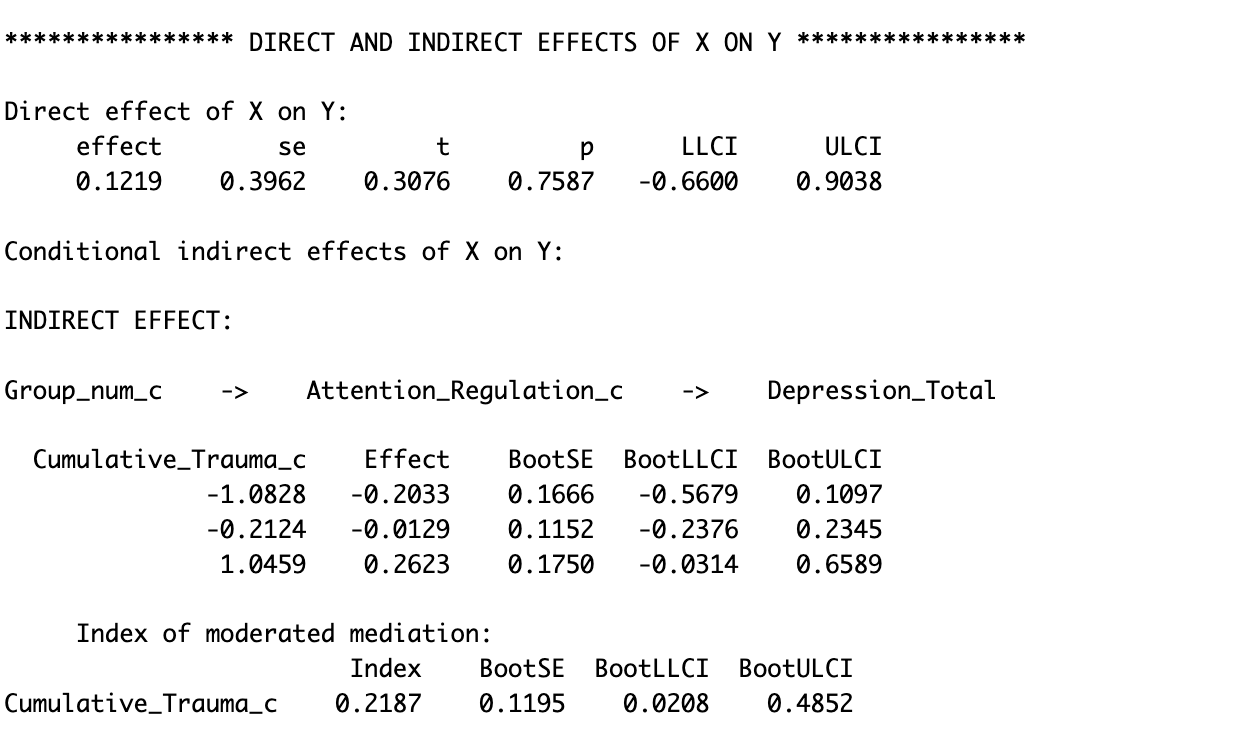


**Supplemental Table 13.** Self-Reg Mod-Med


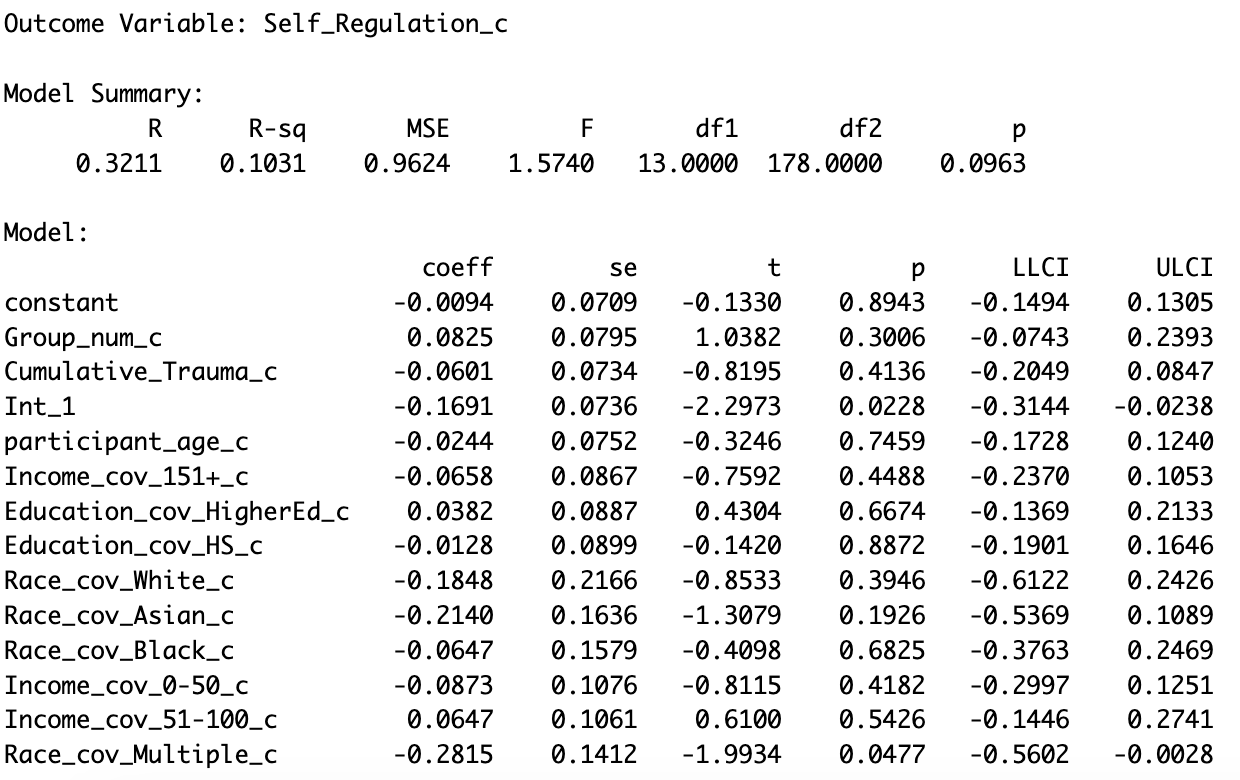


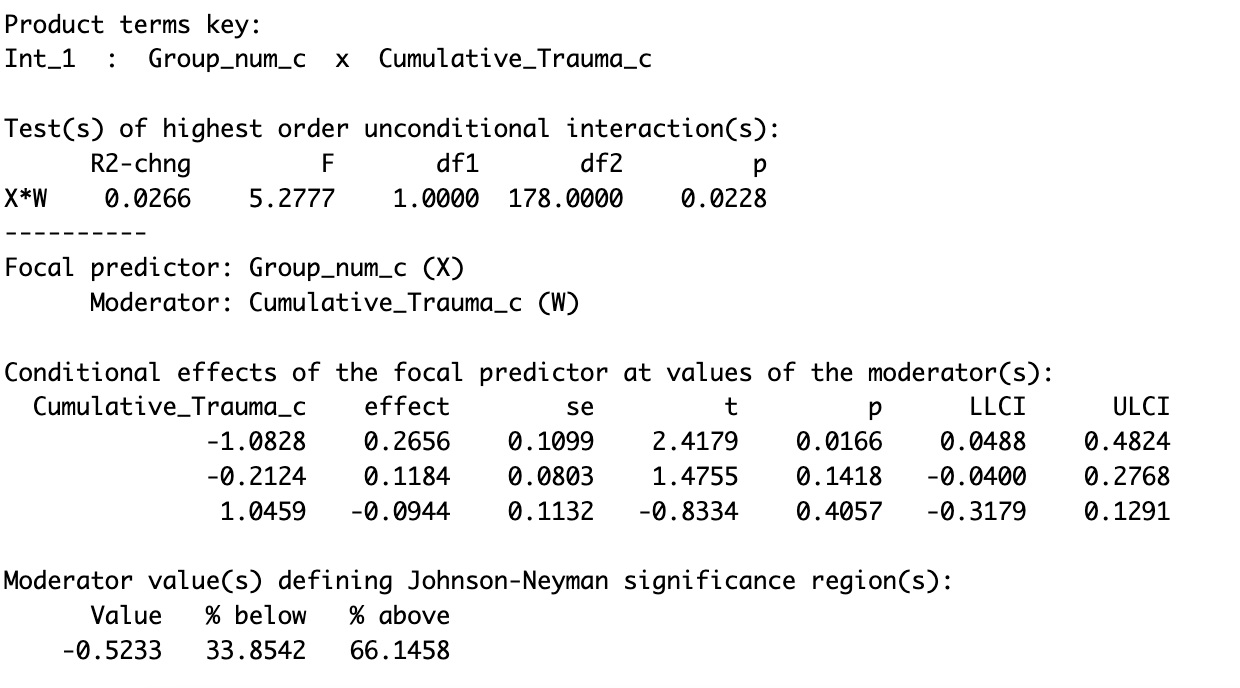


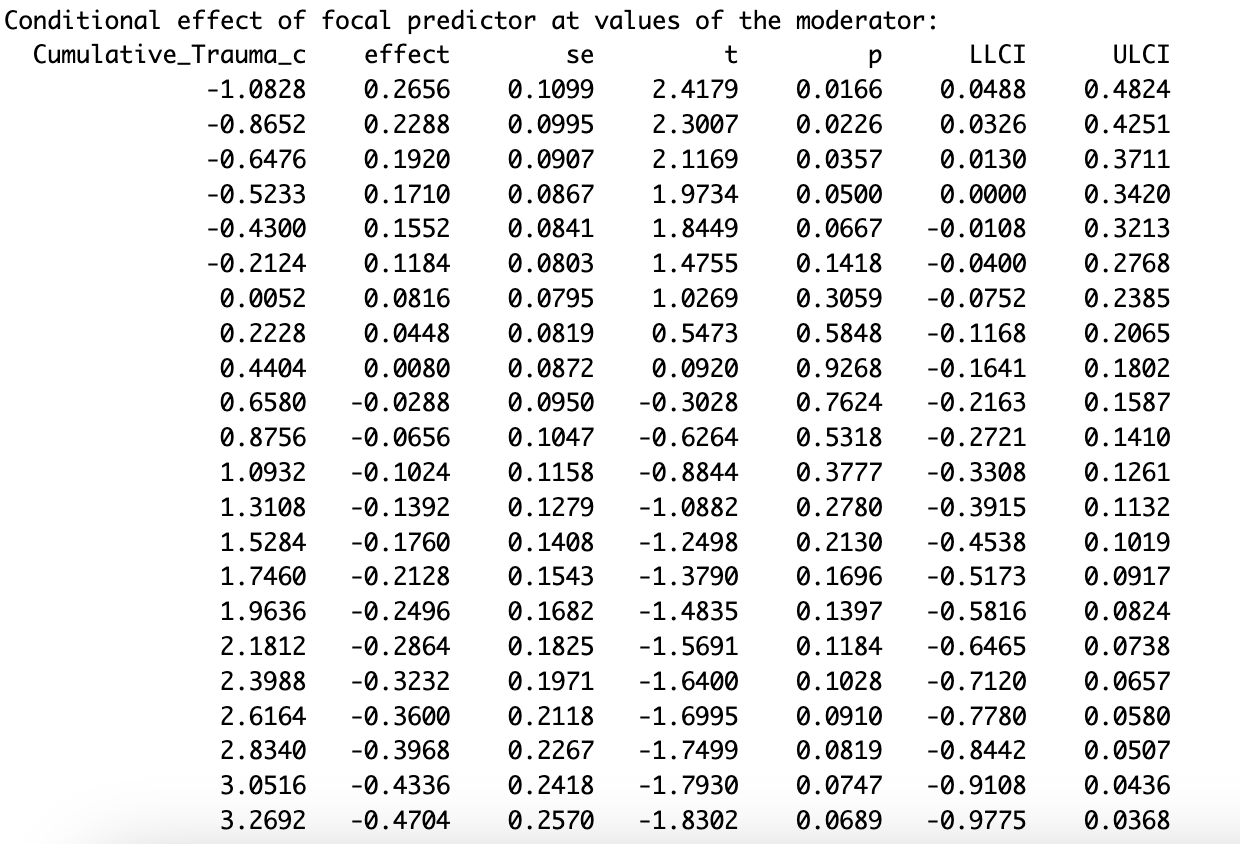


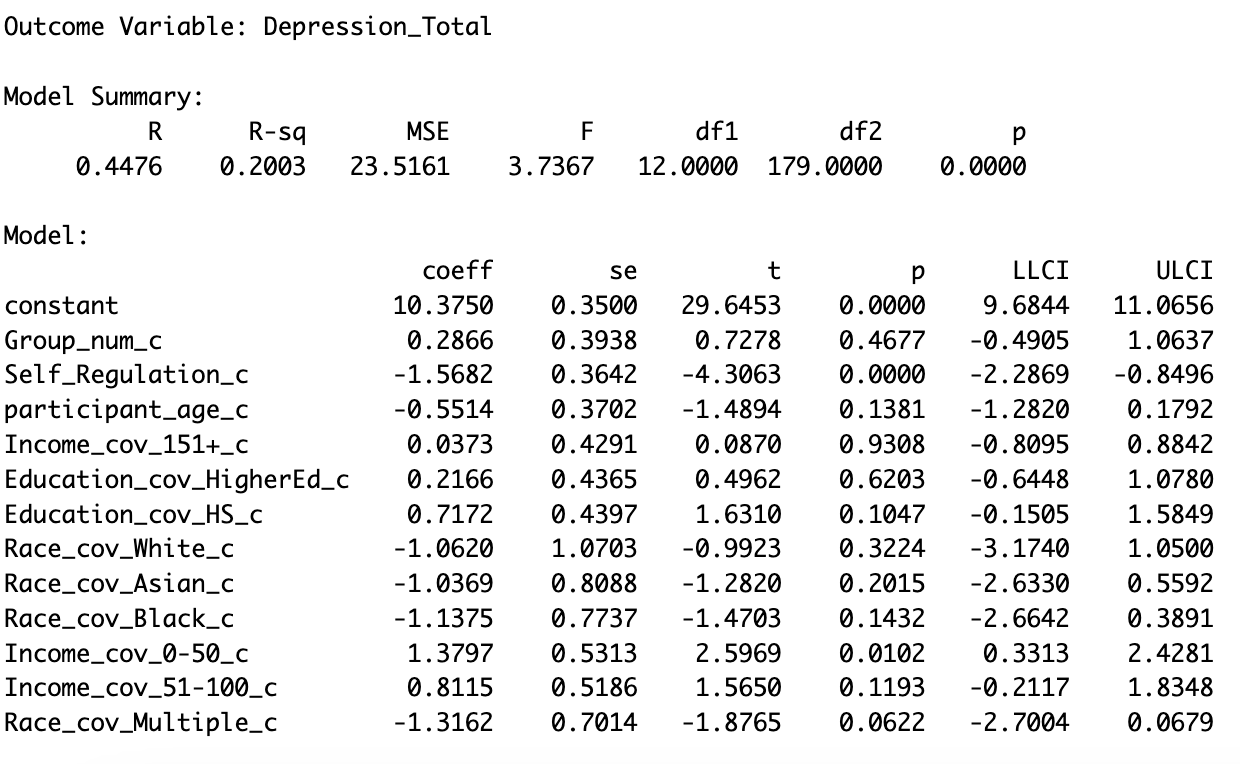


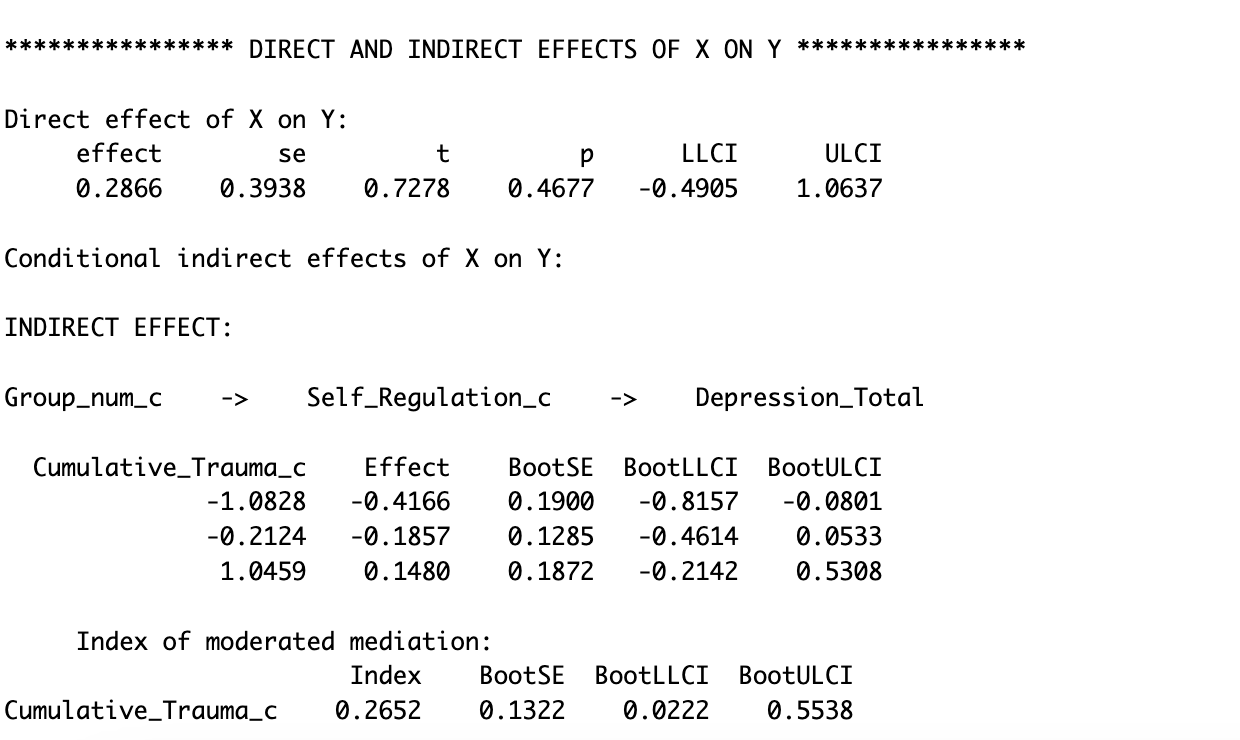


**Supplemental Table 14.** Noticing Mod-Med


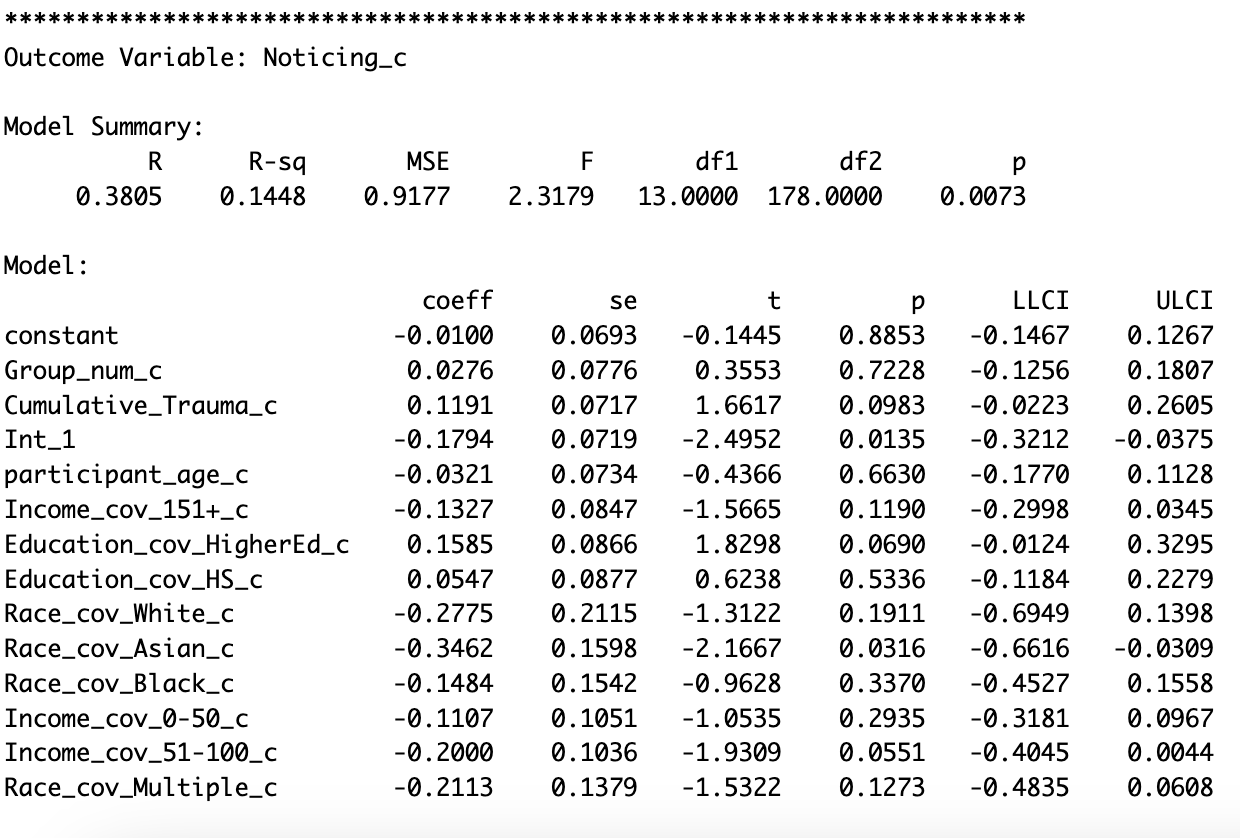


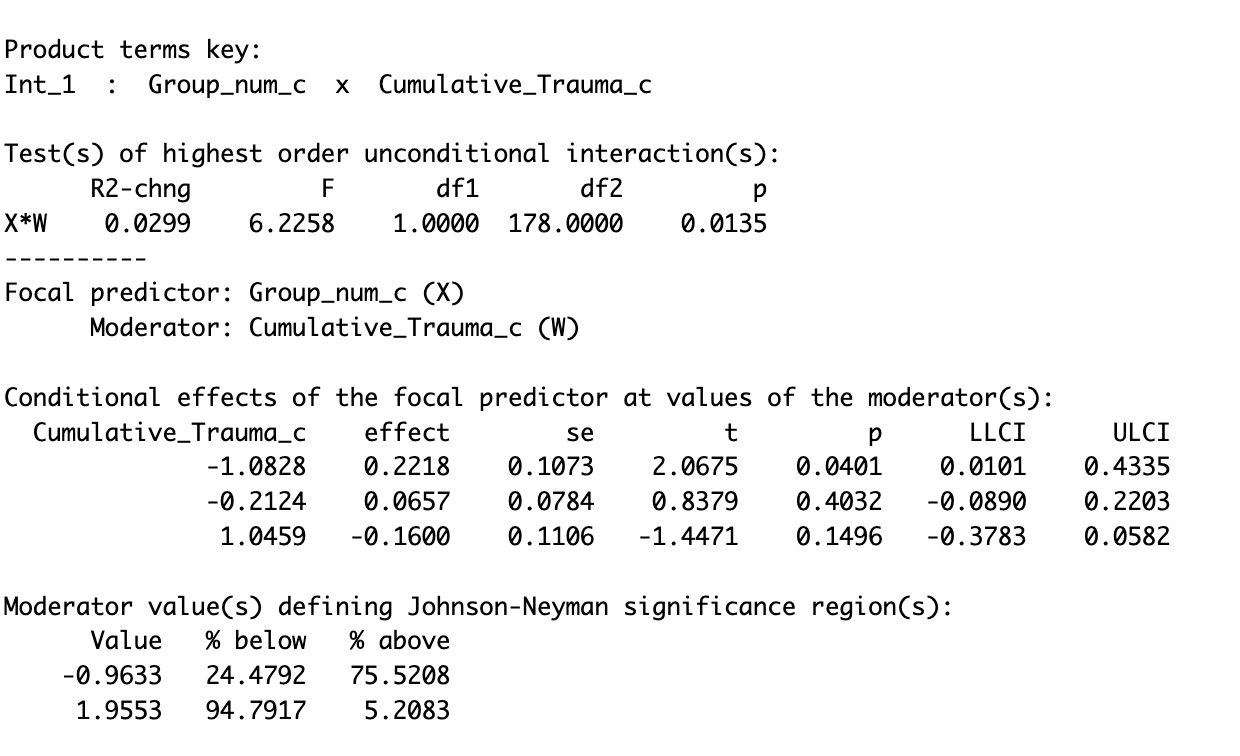


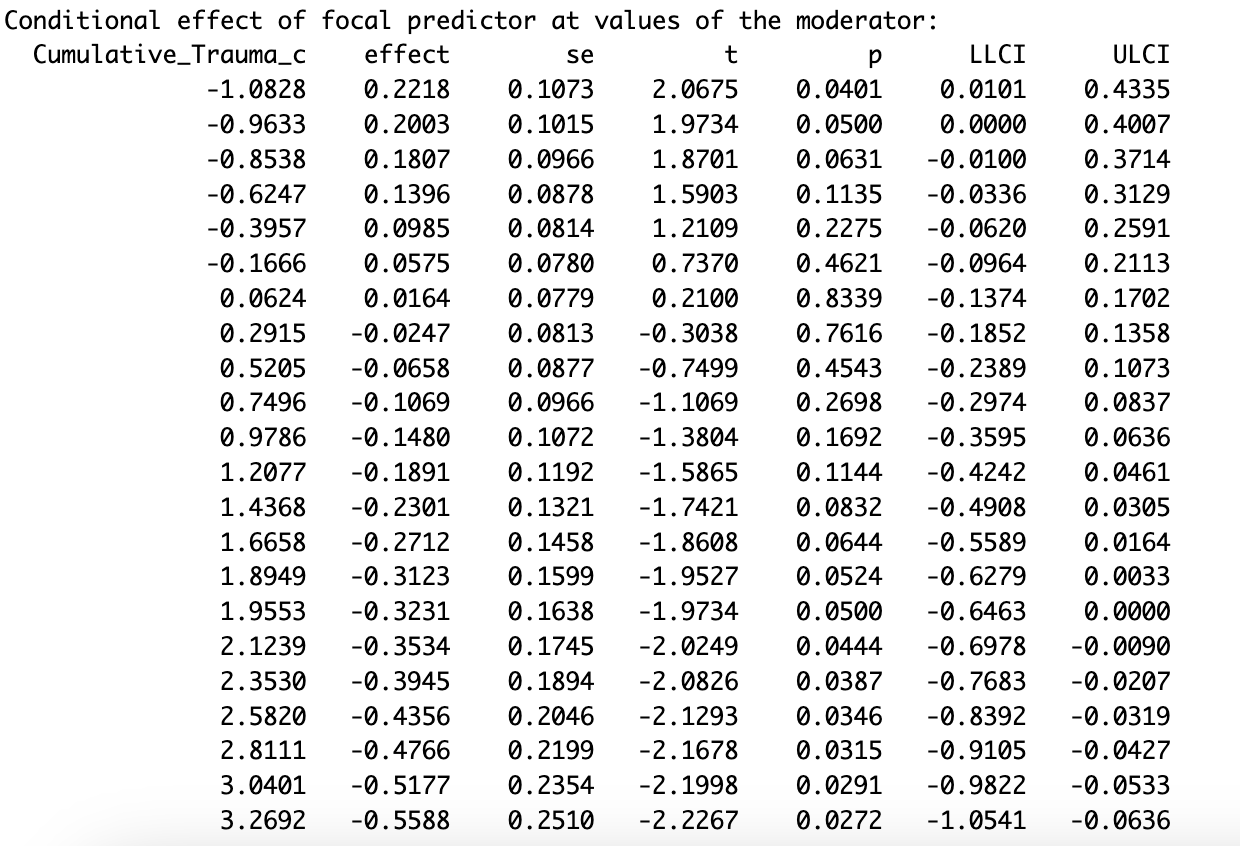


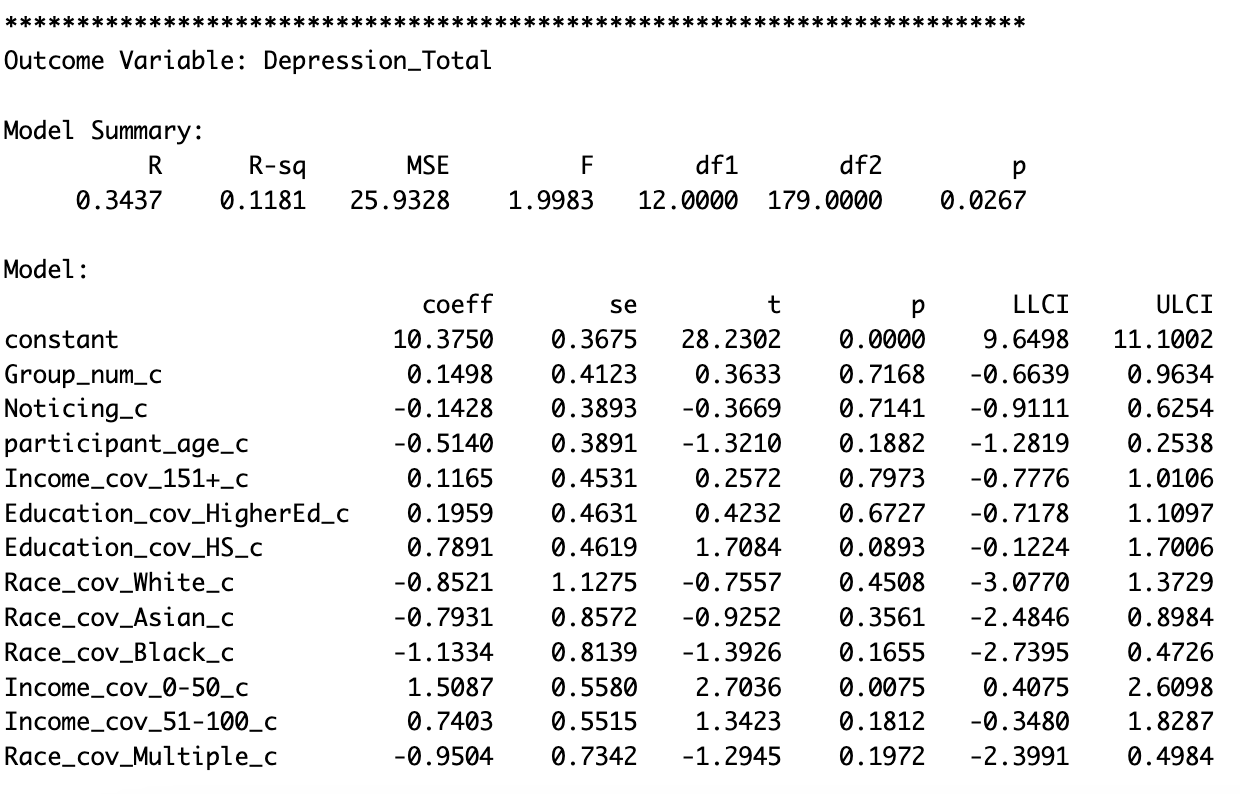


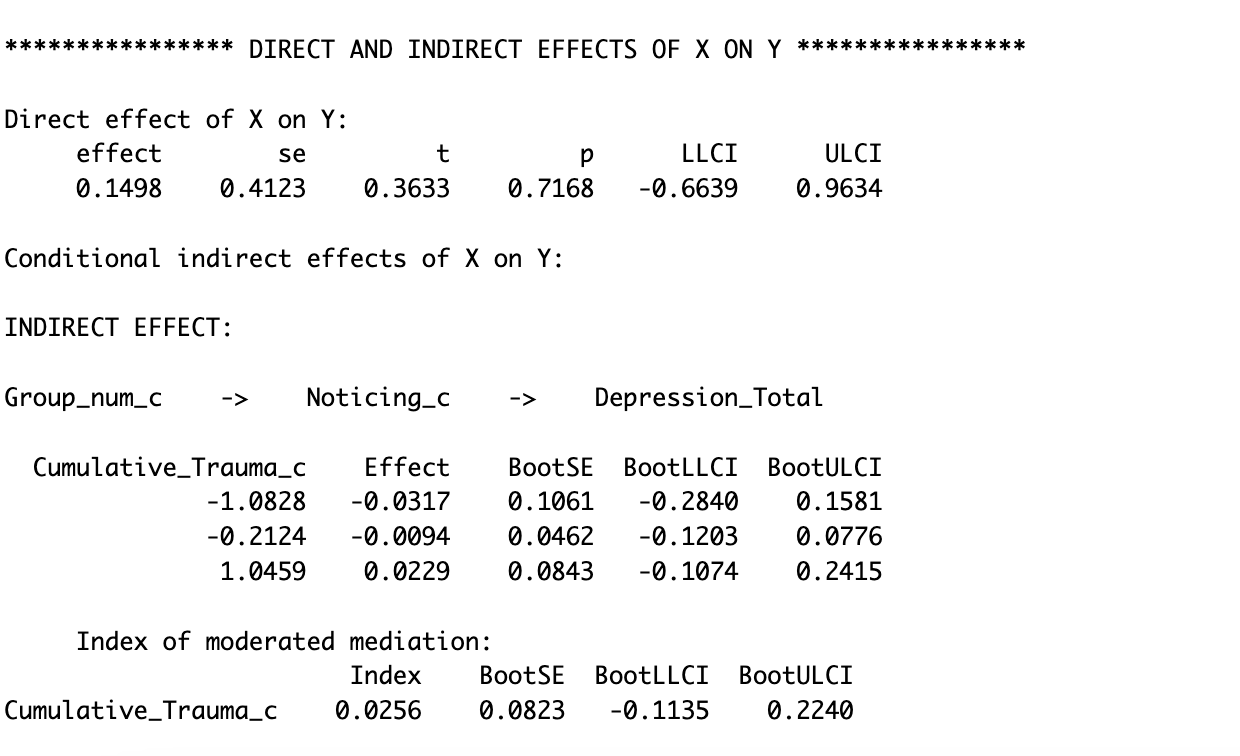

Supplement: Supplementary Material [file NIHMS2034620-supplement-Supplementary_Material.docx]
